# Serum pro-BDNF/BDNF as a treatment biomarker for response to docosahexaenoic acid in traumatized people vulnerable to developing psychological distress: a randomized controlled trial

**DOI:** 10.1038/tp.2015.89

**Published:** 2015-07-07

**Authors:** Y Matsuoka, D Nishi, Y Tanima, M Itakura, M Kojima, K Hamazaki, H Noguchi, T Hamazaki

**Affiliations:** 1 Department of Psychiatry, National Disaster Medical Center, Tachikawa, Japan; 2 CREST, Japan Science and Technology Agency, Kawaguchi, Japan; 3 Health Research Institute, National Institute of Advanced Industrial Science and Technology, Ikeda, Japan; 4 Department of Biochemistry, Kitasato University School of Medicine, Sagamihara, Japan; 5 Department of Public Health, Faculty of Medicine, University of Toyama, Toyama, Japan; 6 Toyama Jonan Onsen Daini Hospital, Toyama, Japan

## Abstract

Our open-label pilot study showed that supplementation with docosahexaenoic acid (DHA) increased serum brain-derived neurotrophic factor (BDNF) levels and that there might be an association between changes in serum BDNF levels and reduced psychological distress. Animal research has indicated that a DHA-enriched diet increases BDNF in the brain. In this randomized double-blind controlled trial of severely injured patients vulnerable to posttraumatic stress disorder (PTSD) and depression, we examined whether DHA increases serum BDNF levels and whether changes in BDNF levels are associated with subsequent symptoms of PTSD and depression. Patients received 1470 mg per day of DHA plus 147 mg per day of eicosapentaenoic acid (EPA; *n*=53) or placebo (*n*=57) for 12 weeks. Serum levels of mature BDNF and precursor pro-BDNF at baseline and 12-week follow-up were measured using enzyme-linked immunosorbent assay kits. At 12 weeks, we used the Clinician-Administered PTSD Scale to assess PTSD symptoms and depressive symptoms by the Montgomery–Åsberg Depression Rating Scale. We found a significant increase in serum BDNF levels during the trial in the DHA and placebo groups with no interaction between time and group. Changes in BDNF levels were not associated with PTSD severity but negatively associated with depression severity (Spearman's *ρ*=−0.257, *P*=0.012). Changes in pro-BDNF were also negatively associated with depression severity (Spearman's *ρ*=−0.253, *P*=0.013). We found no specific effects of DHA on increased serum levels of BDNF and pro-BDNF; however, evidence in this study suggests that increased BDNF and pro-BDNF have a protective effect by minimizing depression severity.

## Introduction

Brain-derived neurotrophic factor (BDNF) has key roles in neuronal differentiation and growth, synapse formation and plasticity, and higher cognitive functions.^1^ The neurotrophin hypothesis of depression states that reduced levels of brain BDNF are associated with depression.^2, 3^ In the early 21st century, low serum BDNF concentrations in depressive patients^4^ and increased BDNF concentrations during the course of antidepressant treatment^5^ were reported. The most recent meta-analysis confirmed that serum BDNF concentrations were low in untreated depressive patients and normalized by antidepressant treatment. However, the meta-analysis highlighted considerable unexplained between-study heterogeneity in outcomes, underpowered study designs and publication bias.^6^ Furthermore, it was suggested that low peripheral BDNF levels were a state biomarker of disease activity reflecting the pathophysiology common to mood disorder and schizophrenia.^7^ BDNF might become a valuable treatment biomarker if early changes in BDNF can be detected during the course of preventing event-related psychological distress. Like other growth factors, BDNF is synthesized from a precursor protein (pro-BDNF). Recent reports have shown that pro-BDNF promotes neuronal death, spine retraction and hippocampal long-term depression,^8, 9, 10^ suggesting that pro-BDNF and BDNF exert opposing biological functions. More recently, hippocampal long-term depression was found to be facilitated in pro-BDNF knock-in mice,^11^ revealing the role of pro-BDNF *in vivo*.

According to experimental studies, omega-3 polyunsaturated fatty acids (PUFAs) can increase the level of brain BDNF^12, 13^ and pro-BDNF.^14^ Omega-3 PUFAs might increase cAMP response element binding protein levels by inhibiting prostaglandin E2 and interleukin-1β, thereby activating BDNF expression.^15^ Energy-generating metabolic pathways are also activated by PUFAs and subsequently affect BDNF.^16^ These findings indicate that the effect of BDNF-related synaptic plasticity, which might be associated with psychological distress, is enhanced by dietary supplementation of omega-3 PUFAs.

Our preliminary open study found that preventive supplementation with docosahexaenoic acid (22:6*n*−3, DHA) increased serum BDNF levels among the injured and that there might be an association between changes in BDNF levels and reduced posttraumatic distress.^17^ We recently completed the first randomized clinical trial to examine the efficacy of DHA for preventing posttraumatic stress disorder (PTSD) and found no significant differences in clinical response as a primary end point.^18^ This secondary analysis aimed to investigate the following: (1) the effects of omega-3 DHA on serum levels of pro-BDNF and mature BDNF in injured patients admitted to the intensive care unit immediately following accidental injury, and (2) the association between changes in serum levels of pro-BDNF and mature BDNF and psychological distress such as PTSD and major depressive disorder.

## Materials and methods

### Design

This study was a randomized double-blind, placebo-controlled omega-3 PUFA prevention trial (trial registration: Clinicaltrials.gov Identifier: NCT00671099). All patients received psychoeducation and were randomly assigned in a 1:1 manner to receive DHA-enriched capsules or placebo capsules for 12 weeks. The Ethics Committee of the National Disaster Medical Center approved this study and all participants provided written informed consent. Full trial protocol can be available in a previous publication.^19^

### Participants

We recruited 110 injured patients aged 18–82 years treated in the intensive care unit of the National Disaster Medical Center from 16 December 2008 to 6 June 2013. The following inclusion criteria were applied: (1) ⩾18 years of age; (2) native speaker of Japanese; (3) recruited within 240 h after injury; and (4) physically and psychologically able to understand the present trial's scope and to provide written consent for participation in the study. The exclusion criteria were as follows: (1) obviously irreversible acute brain parenchymal damage, or subdural or subarachnoid bleeding; (2) Mini-Mental State Examination score <24;^20^ (3) a serious drinking problem; (4) a smoking habit of ⩾40 cigarettes per day; (5) history and suspicion of psychosis or bipolar I disorder; (6) suspicion of alcohol- or substance-related disorder or eating disorder; (7) suicidal ideation, self-harm behavior, severe dissociation or other serious psychiatric condition; (8) regular treatment with anti-epilepsy medication, lithium, ethyl-icosapentate, aspirin or warfarin within the last 3 months; (9) regular pre-accident consumption of PUFA supplements within the last 3 months; and (10) a habit of eating fish ⩾4 times per week.

**Figure 1:**
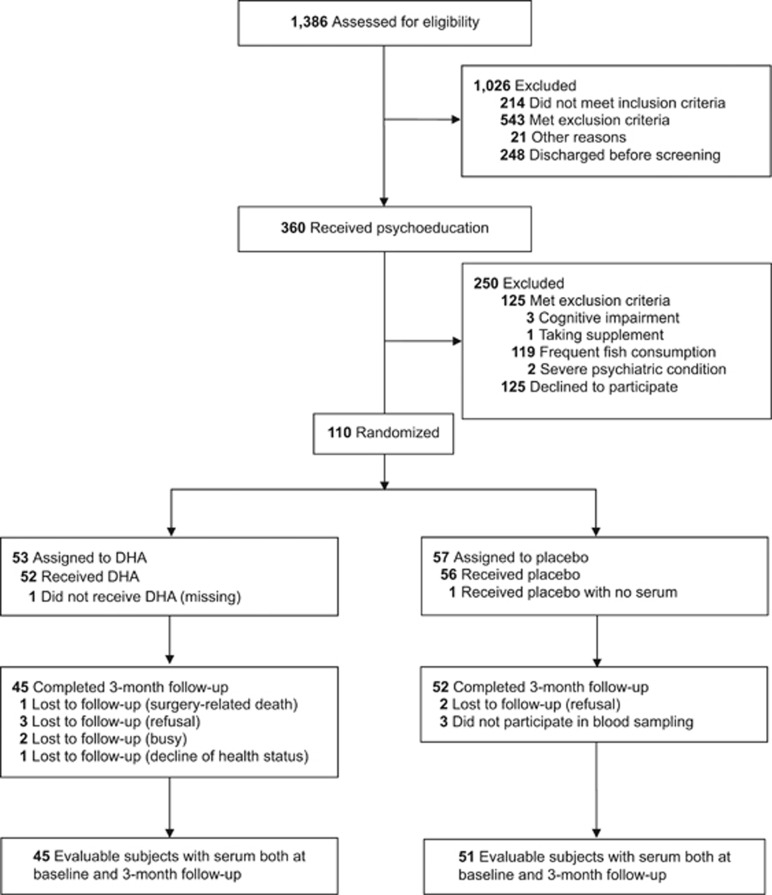
Flow diagram of the present study. DHA, docosahexaenoic acid.

Figure 1 presents the participant flow during the trial. Baseline samples for determination of erythrocyte membrane DHA were available from 110 participants. The trauma types in participants were broken bones (60%), head injury (17.3%), spinal cord injury (13.6%), visceral injuries (6.4%) and others (2.7%). We collected follow-up erythrocyte samples at the end of intervention from 97 individuals. Baseline serum samples for determination of BDNF and pro-BDNF were available from 109 participants. Follow-up serum samples were collected from 97 individuals. Table 1 shows the baseline characteristics, erythrocyte DHA and serum BDNF at weeks 0 and 12.

### Treatment interventions

The active treatment was a dietary supplement of dark brown gelatin capsules (300 mg) containing concentrated marine fish oil. The daily dose was seven capsules, which contained a total of 1470 mg DHA and 147 mg eicosapentaenoic acid (EPA; 20:5*n*−3), and 0.3% alpha-tocopherol. A mixture of rapeseed oil (47%), soybean oil (25%), olive oil (25%), fish oil (3%) and 0.3% α-tocopherol was used as the control oil. The control oil had a fatty acid composition similar to average composition of fatty acid intake in Japan. A small amount of not-fully deodorized fish oil was added to the control oil to give it a fishy odor/taste so that it could not be distinguished from the active oil.

### Psychological assessments

The Clinician-Administered PTSD Scale (CAPS),^21, 22^ the Montgomery–Åsberg Depression Rating Scale (MADRS),^23, 24^ the Peritraumatic Distress Inventory^25, 26^ and the CD-RISC (Conner–Davidson Resilience Scale) were used for psychological assessments.^27^ The primary outcome measure was the CAPS total score at the 12-week follow-up. More details on these psychological assessments are available in a previous publication.^19^

### Analysis of erythrocyte DHA composition

At baseline (that is, time point before intensive care unit admission) and at the 12-week follow-up (end of intervention), erythrocyte DHA composition was quantified. Bligh and Dyer's method was used to extract total lipids.^28^ Total phospholipid fractions were separated by preparative thin-layer chromatography. Transmethylation was performed using HCl–methanol, and then fatty acid composition was analyzed using a gas chromatography system (GC-2014, Shimadzu, Kyoto, Japan) equipped with a DB-225 capillary column (length, 30 m; internal diameter, 0.25 mm; film 0.25 μm; J&M Scientific, Folsom, CA, USA). The entire system was controlled using GCsolution version 2.3 (Shimadzu). We used means (expressed as percentage of total fatty acids) for peak DHA to index pre- to posttreatment fatty acid composition of the erythrocyte membrane as an objective measure of treatment adherence.

### Analysis of serum BDNF and pro-BDNF

To assess the serum level of BDNF and pro-BDNF, 7–10 ml of blood was drawn at week 0 and week 12 and serum samples were stored at −80 °C until analysis. Serum BDNF levels were measured with the BDNF Emax Immunoassay system kit (Catalog (Cat.) #G7611, Promega, Madison, WI, USA) according to the manufacturer's instructions. The analyst was masked to the subjects' diagnostic status when performing the BDNF and pro-BDNF ELISA (enzyme-linked immunosorbent assay) assays.

For BDNF ELISA, 96-well plates were coated with anti-BDNF monoclonal antibody and incubated at 4 °C for 18 h. The plates were next washed with Tris-buffered saline containing Tween 20 (TBST; 20 mM Tris-HCl, 150 mM NaCl, 0.05% Tween 20). The plates were incubated in a blocking buffer for 1 h without shaking and then washed. The samples and BDNF standards were shaken for 2 h and then washed. The plates were incubated with anti-human BDNF polyclonal antibody for 2 h with shaking, washed and incubated with anti-IgY antibody conjugated to horseradish peroxidase for 1 h at room temperature and then washed. For colorimetric analysis, the plates were incubated with peroxidase substrate and tetramethylbenzidine solution. After stopping the reaction by the addition of hydrochloric acid (1 M), the absorbance at 450 nm was measured with an Emax automated microplate reader (Molecular Devices, Tokyo, Japan). Following the incubation at 4 °C, all the operations were performed at room temperature.

Serum levels of pro-BDNF were similarly determined by ELISA. Anti-pro-BDNF monoclonal antibody was used as the first antibody. The antibody was produced by the following method. A synthetic peptide corresponding to human pro-BDNF amino-acid residues 69–82 ((C)ELLDEDQKVRPNEE) was conjugated to maleimide-activated keyhole limpet hemocyanin (Pierce, Rockford, IL, USA) and injected into rabbits. The antiserum was affinity-purified using the antigenic peptide coupled to epoxy-activated sepharose 6B (GE Healthcare Life Science, Buckinghamshire, UK). Recombinant pro-BDNF (Wt-human; Cat. #B-257, Alomone Labs, Jerusalem, Israel) was used as the standard protein in this assay. Briefly, 96-well ELISA plates were coated with the anti-pro-BDNF monoclonal antibody and incubated at 4 °C for 18 h and then washed once with TBST. After incubation with a blocking buffer (Cat. #G3311, Promega) for 1 h, the samples and recombinant pro-BDNF (Wt-human; Cat. #B-257, Alomone Labs) were applied. After incubation for 2 h at room temperature, the ELISA plates were washed five times with the TBST buffer. The second antibody used was chicken polyclonal antibody against human BDNF (Cat. #G1641, Promega). After incubation for 2 h at room temperature, the ELISA plates were washed five times with TBST buffer. Anti-IgY antibody conjugated to horseradish peroxidase was applied and the plates were incubated for 1 h at room temperature. The plates were washed with TBST buffer and incubated in peroxidase substrate and tetramethylbenzidine solution for 10 min for colorimetric analysis. Hydrochloric acid (1 M) was added to stop the reaction and the absorbance at 450 nm was measured with an Emax automated microplate reader (Molecular Devices).

### Randomization and blinding

An independent statistician generated randomization lists with three stratification factors using a computer-generated random allocation sequence by block-randomization method. Stratification factors included sex, age (<40 or ⩾40 years), and sense-of-life threat (yes or no). The randomization list was sent to an independent pharmacist who securely kept the tables and prepared numbered supplement bottles according to the list. An allocation Excel sheet file was masked and securely kept under passcode by the pharmacist. Both the research team and participants were masked to randomization until the last participant completed the protocol and the spreadsheets of all the results were finalized.

### Statistical analysis

To the cross-sectional comparison of the DHA group and placebo group at baseline, we applied the chi-square test or Student's *t*-test or Mann–Whitney *U*-test. Serum levels of BDNF appeared normally distributed, whereas those of pro-BDNF did not. We converted pro-BDNF data to logarithmic values, but the distribution remained non-normal. We performed repeated measures analysis of variance with time (baseline vs follow-up) as a within-subject factor to compare BDNF serum levels between the two groups (DHA vs placebo). For reference, we performed a similar repeated measures analysis of variance for pro-BDNF. To examine associations between serum pro-BDNF/BDNF and psychopathology, correlation analysis was performed. Spearman's correlation coefficients *ρ* were calculated between changes in BDNF and psychopathology (CAPS total score, MADRS score, CD-RISC score) at the 12-week follow-up for the whole group. In addition, to exclude the effect of DHA, Spearman's correlation coefficients were calculated between changes in BDNF and psychopathology at the 12-week follow-up in the control group only. We performed all the analyses using SPSS version 22.0 J for Windows (SPSS, Tokyo, Japan). All the tests were two-sided, and *P*-values of 0.05 or less were considered statistically significant.

For sample-size estimation, at least 49 cases per group were required given that the expected difference in CAPS score between the groups at 3 months as a primary end point was set at 10 (s.d.=15) with an alpha level of 0.05 (two-tailed) and 90% power on the basis of our open pilot study^29^ and some previous studies of omega-3 PUFA on depressive disorder. As this is an exploratory secondary analysis, we did not estimate sample size for BDNF study.

## Results

Table 1 shows the baseline characteristics of the sample. At baseline, the two groups did not differ in demographic variables, clinical characteristics, body mass index, or erythrocyte DHA composition. Adherence to the preventive intervention seemed to be good because erythrocyte DHA composition at the 12-week follow-up in the DHA group was significantly higher than that in the placebo group (Table 1). Table 1 presents mean levels of serum BDNF and pro-BDNF for each treatment group at baseline and at the 12-week follow-up. Repeated measures analysis of variance showed a significant main effect for time (baseline vs 12-week follow-up, F=62.30, df=1,94, *P*<0.001), but no significant main effect for group (DHA vs placebo, F=0.20, df=1,94, *P*=0.65) and time-by-group interaction (F=0.78, df=1,94, *P*=0.38) in serum BDNF levels. Repeated measures analysis of variance showed no significant main effect for time (F=2.25, df=1,94, *P*=0.14), group (F=3.07, df=1,94, *P*=0.08) and time-by-group interaction (F=0.90, df=1,94, *P*=0.34) in serum pro-BDNF levels.

Changes in serum BDNF and pro-BDNF in the whole sample were negatively correlated to the MADRS score (Table 2). However, we found no significant correlation between changes in serum BDNF or pro-BDNF and either CAPS or CD-RISC score. In addition, a significant negative correlation was found between changes in serum BDNF and the MADRS score in the placebo group. In contrast, unfortunately, we found no significant correlation between changes in serum BDNF and the MADRS score in the DHA group.

## Discussion

In this randomized controlled trial, we observed increases in serum BDNF levels in injured patients after they received either DHA or placebo compared with baseline. We found no specific effect of DHA on serum BDNF and pro-BDNF levels in patients with accidental injury and no psychotropic regular medication. Changes in serum BDNF and pro-BDNF levels at week 12 were inversely associated with depression severity. The association between changes in serum BDNF levels and depression severity remained significant in the placebo group alone. Our findings suggest that using early increases in serum BDNF as a treatment biomarker in future investigations might be useful for identifying individuals resilient to major depressive disorder after a traumatic event.

Unfortunately, we could not detect a specific effect of DHA on serum levels of BDNF and pro-BDNF in this trial. As 12-week supplementation regardless of content should have an impact on serum BDNF levels, our result was unexpected. Two earlier meta-analyses^30, 31^ included studies that evaluated whether BDNF levels were associated with improvement of depression, but those studies were all uncontrolled trials. Although they reported significant increases in BDNF levels after antidepressant treatment, the possibility of a time effect cannot be ruled out. Compared with previous antidepressant studies, the present study has a higher level of evidence due to its study design. The association between improvement of depression in previous studies and minimization of developing depression in the present study and increased serum BDNF might reflect intervention time effects.

Increased levels of serum BDNF and pro-BDNF after a traumatic event were not associated with PTSD symptoms and resilience. In our previous cohort study of motor vehicle accident survivors, a positive correlation was found between changes in serum BDNF levels over a 6-month period and the CAPS score at 6 months.^32^ After checking the correlation between changes in serum BDNF levels during 1 month and the CAPS score at 1 month in our previous cohort study,^32^ we did not find any correlation at all (*ρ*=−0.042, *P*=0.65, unpublished data). These inconsistent results in relation to the present study might be explained by assessment time (1–3 months vs 6 months). Berger *et al.*^33^ reported that despite a substantial decrease in PTSD and comorbid depressive symptoms over the course of an open-label trial of escitalopram monotherapy, they detected no significant changes in serum BDNF levels throughout the 12-week therapy period. Our pilot trial of DHA supplementation for preventing accident-related PTSD showed an association between increased serum BDNF levels and prevention of PTSD and major depressive disorder.^17^ Therefore, whether serum BDNF affects PTSD symptoms remains unclear. However, it is important to note that changes in serum BDNF and pro-BDNF during the trial were inversely correlated with depression severity at follow-up in the whole sample, although we do not understand the precise mechanism involved in the post-supplementation elevation in serum BDNF.

The characteristics of our study include a randomized double-blind, placebo-controlled design, use of reliable assessments, an objective measure of treatment adherence and a low attrition rate. However, a limitation should be noted. First, we obtained our results solely from Japanese patients who frequently consumed seafood. Omega-3 PUFA intake in Brazilian adolescents (estimated by food frequency questionnaire) was positively associated with serum BDNF levels.^34^ More studies are needed to elucidate the effect of DHA on serum BDNF including those from different parts of the world. Second, the traumatic event included only severe accidental injury. Other types of traumatic events might affect PTSD and major depressive disorder differently.

Although we found no effect of DHA on prevention of PTSD or serum BDNF levels, the present study underscores the need to reconsider the implications of the neurotrophin hypothesis of depression. In conclusion, our data suggest that changes in serum BDNF regardless of any treatment condition might be a central phenomenon in improving or minimizing depressive symptoms.

## Figures and Tables

**Table 1:** Baseline characteristics, serum BDNF levels (ng ml^−1^) and DHA composition (%) at baseline and at 12-week follow-up by intervention group

	*DHA (*n=*53)*	*Placebo (*n=*57)*	P
Men	44 (83)	46 (81)	0.75
			
*Type of injury*			
Traffic accident	40 (75.5)	43 (75.4)	0.99
Falling from height	9 (17.0)	10 (17.5)	
Workplace accident and other	4 (7.5)	4 (7.0)	
Mild traumatic brain injury	28 (52.8)	20 (35.1)	0.06
			
Age (years)	38.1±13.5	40.9±17.3	0.34
Glasgow coma scale	14.6±1.2	14.8±0.5	0.22
Injury severity scale	9.6±6.4	9.0±4.8	0.59
Body mass index	22.9±3.7	23.4±4.4	0.53
Education (years)	13.1±1.9	12.4±2.4	0.10
Peritraumatic distress inventory	13.7±10.3	13.7±8.4	0.99
Erythrocyte DHA at baseline (%)^a^	6.4±1.3	6.3±1.3	0.65
Erythrocyte DHA at 12 weeks (%)^b^	8.9±1.1	6.7±0.9	<0.01
Change in DHA during 12 weeks (%)^b^	2.5±1.5	0.4±0.7	<0.01
Serum BDNF levels at baseline (ng ml^−1^)^c^	16.0±5.2	16.4±5.1	0.67
Serum BDNF levels at 12 weeks (ng ml^−1^)^b^	20.0±4.1	20.0±4.4	0.94
Serum pro-BDNF levels at baseline (ng ml^−1^)^c^	23.4±47.9	12.4±12.5	0.10
Median (range)	9.0 (2.8–273.8)	7.9 (1.5–66.6)	0.37
Serum pro-BDNF levels at 12 weeks (ng ml^−1^)^b^	34.7±83.1	14.8±18.4	0.10
Median (range)	11.0 (3.9–483.4)	9.2 (3.2–122.0)	0.22

Abbreviations: BDNF, brain-derived neurotrophic factor; DHA, docosahexaenoic acid.

a DHA group: *n*=53; placebo group: *n*=57.

b DHA group: *n*=45; placebo group: *n*=52.

c DHA group: *n*=53; placebo group: *n*=56.

Data are expressed as mean±s.d. or as values with percentages in parenthesis.

**Table 2:** Correlations between the changes in BDNF levels between baseline and 12-week follow-up (ng ml^−1^) and psychopathology at 12-week follow-up

	*Changes in BDNF*			*Changes in pro-BDNF*		
	*All* (n=97)	*DHA* (n=46)	*Placebo* (n=51)	*All* (n=97)	*DHA* (n=46)	*Placebo* (n=51)
*PTSD severity*						
CAPS total score	−0.177	−0.284	−0.053	−0.164	−0.219	−0.086
						
*Depression severity*						
MADRS total score^a^	−0.257*	−0.192	−0.298*	−0.253*	−0.257	−0.251
						
*Resilience*						
CD-RISC total score^a^	0.091	0.249	−0.042	0.062	−0.042	0.191

Abbreviations: BDNF, brain-derived neurotrophic factor; CAPS, Clinician-Administered PTSD Scale; CD-RISC, Connor–Davidson Resilience Scale; DHA, docosahexaenoic acid; MADRS, Montgomery–Åsberg Depression Rating Scale; PTSD, posttraumatic stress disorder.

a *n*=96 in both groups.

*P<0.05.

## Notes

YM has received research grants from the Japan Science and Technology Agency, National Center of Neurology and Psychiatry Japan, and the Ministry of Health, Labour, and Welfare of Japan; and has been a paid speaker for Ono Pharmaceutical, Mochida Pharmaceutical, Takeda Pharmaceutical Company, Suntory Wellness, Otsuka Pharmaceutical and the DHA & EPA Association. DN has received research grants from the Japan Society for the Promotion of Science, lecture fees from Qol, the DHA & EPA Association, NTT DoCoMo and Emotional Quotient Academy. MK has received research grants from the Japan Science and Technology Agency. MI has received research grants from the Japan Science and Technology Agency. KH has received research support from the National Center of Neurology and Psychiatry, the Japan Society for the Promotion of Science, the Tamura Foundation for Promotion of Science and Technology and the Ichiro Kanehara Foundation for Promotion of Medical Sciences and Medical Care; has received consultant fees from Polyene Project; has received scholarship donations from Otsuka Pharmaceutical; and has been a paid speaker for the DHA & EPA Association. TH has received research support from the Japan Society for the Promotion of Science, Open Research Center for Lipid Nutrition (Kinjo Gakuin University) and Nippon Suisan Kaisha; has received consultancy fees from Polyene Project and Otsuka Pharmaceutical; and has received lecture fees from Otsuka Pharmaceutical and travel expenses from Aker BioMarine. The remaining authors declare no conflict of interest.
